# Role of water in unexpectedly large changes in emission flux of volatile organic compounds in soils under dynamic temperature conditions

**DOI:** 10.1038/s41598-022-08270-5

**Published:** 2022-03-15

**Authors:** Asma Akter Parlin, Monami Kondo, Noriaki Watanabe, Kengo Nakamura, Jiajie Wang, Yasuhide Sakamoto, Takeshi Komai

**Affiliations:** 1 Department of Environmental Studies for Advanced Society, Graduate School of Environmental Studies, Tohoku University, Sendai, 9808579 Japan; 2 National Institute of Advanced Industrial Science and Technology (AIST), Tsukuba, 3058567 Japan

**Keywords:** Environmental sciences, Atmospheric science

## Abstract

Understanding the diffusive transport behavior of volatile organic compounds (VOCs) in near-surface soils is important because soil VOC emissions affect atmospheric conditions and climate. Previous studies have suggested that temperature changes affect the transport behavior; however, the effect of these changes are poorly understood. Indeed, under dynamic temperature conditions, the change in VOC flux is much larger than that expected from the temperature dependency of the diffusion coefficient of VOCs in air. However, the mechanism is not well understood, although water in soil has been considered to play an important role. Here, we present the results of experiments for the upward vertical vapor-phase diffusive transport of two VOCs (benzene and tetrachloroethylene) in sandy soil under sinusoidal temperature variations of 20–30 °C, as well as its numerical representation. The results clarify that the unexpectedly large changes in emission flux can occur as a result of changes in the VOC concentration gradient due to VOC release (volatilization) from/trapping (dissolution) into water, and that such flux changes may occur in various environments. This study suggests the importance of a global evaluation of soil VOC emissions by continuous measurements in various soil environments and/or predictions through numerical simulations with thorough consideration of the role of water in dynamic soil environments.

## Introduction

The transport behavior of volatile organic compounds (VOCs), such as petroleum and chlorinated hydrocarbons, in near-surface soils has been widely studied because these relatively long-lived VOCs pose a serious environmental threat, owing to their mass production and widespread use in manufacturing^1–3^. VOC vapors can migrate from contaminated near-surface soils into buildings when they underlie building foundations, thereby increasing indoor VOC concentrations above those in outdoor environments^4,5^. Indoor inhalation of VOCs is an important exposure pathway at VOC-contaminated sites and poses a serious risk to human health^6–8^. In contrast, VOCs in soil—especially biogenic VOCs—will eventually be emitted from soils into the atmosphere and possibly participate in atmospheric photochemical reactions that contribute to the formation of secondary compounds, such as ozone and secondary organic aerosols^9,10^. Therefore, VOC emissions from soils may contribute to the mitigation of climate change via formation of aerosols that are also involved in climate feedback processes^11,12^. Consequently, a global evaluation of VOC emissions from soils is of great interest in advancing our knowledge of the impacts of VOC emissions from soils on atmospheric conditions and climate.

Several factors influence VOC vapor transport behavior in near-surface soils. For example, past studies have shown that VOC vapor transport is substantially affected by soil temperature, and in static temperature environments, rates for diffusive transport of VOCs increase with temperature^13–17^. However, due to the dynamic nature of these environments and/or inhomogeneities in the soil properties, transport behavior of VOC vapor can be counterintuitive or unexpected. Field observations of near-surface soils to a depth of 3 m suggested an inverse relationship between vertical VOC flux and soil temperature^13^. The trichloroethylene (TCE) flux was related positively to the temperature at shallower depths, but an inverse relationship was found at deeper depths^13^. Furthermore, the flux changes were larger than those expected based on the temperature dependence of the diffusion coefficient of TCE in air (Supplementary Figure S1a), where the flux could increase by more than twofold and could decline to near-zero or negative values^13^. Such counterintuitive and unexpected flux changes may be linked to the changes in indoor VOC concentrations^18^. Changes in the indoor concentration of tetrachloroethylene (PCE) are closely related to soil temperature at relatively shallow depths in a depth range of approximately 3 m but not with deeper soil temperatures^18^. Moreover, the indoor concentration of PCE could show a twofold increase even with a usual increase in soil temperature (e.g., 5 °C), which was not predicted based on the temperature dependence of the diffusion coefficient of PCE in air (Supplementary Figure S1a).

However, to the best of our knowledge, such inverse relationships and unexpectedly large changes in dynamic temperature conditions have rarely been investigated in laboratory studies. The majority of laboratory experiments concerning gas-phase diffusive VOC transport in soils have been performed at a constant temperature^19–27^. The few laboratory experiments that have considered dynamic conditions in near-surface soils did not cover the unexpected or counterintuitive effects of temperature on VOC flux^28,29^. In this context, our recent studies^30,31^ revealed the inverse relationship between flux and temperature changes and enhanced flux changes. Moreover, enhanced flux changes were assessed based on experiments of upward vertical vapor-phase diffusive transport of benzene and TCE^30,31^. These experiments were conducted in finer and coarser grained sandy soils, with water contents ranging from air-dry to 10 wt% during sinusoidal temperature variation between 20 and 30 °C. In these previous experiments, the flux from the soil surface was related positively to temperature, whereas the flux into the overlying soil exhibited an inverse relationship with temperature. The inverse relationship and enhanced flux changes were clearly observed in all conditions, except in the air-dry condition, in the presence of water condensation/evaporation, and their strengths did not depend on sand grain size but were greater for TCE with stronger temperature dependence of its volatility (e.g., Henry’s constant in Supplementary Figure S1b), higher water content, and/or larger intensity in the phase change of water. It was, therefore, hypothesized that such flux changes are induced by changes in the vertical concentration gradient of VOCs resulting from concentration changes due to VOC release (volatilization) from and trapping (dissolution) into water, and/or water evaporation and condensation that contribute to VOC release and trapping, respectively. However, to the best of our knowledge, this hypothesis has not previously been examined.

Therefore, in the present study we examined this hypothesis using a novel laboratory experiment and a complementary numerical simulation of upward vertical vapor-phase diffusive transport of VOCs in soil under dynamic temperature. We initially describe a new experimental method for exploring the vertical vapor-phase diffusive transport of VOCs in the soil, which has been developed based on the experimental methods in our previous studies^30,31^ and allows continuous measurements of VOC concentrations at multiple depths and of relative humidity (RH) in a soil-packed column under dynamic temperature conditions. Thereafter, an experiment using benzene at a static temperature (25 °C) was conducted to verify that no fluctuations in concentration or RH occurred because of the multiple-depth concentration measurement by gas detectors equipped with a pump. Next, we provide the results of two experiments using benzene and PCE at a dynamic temperature (25 ± 5 °C) to clarify the concentration changes at multiple depths and the resultant concentration gradient, phase change of water, and their relationships to changes in VOC emission flux. Finally, a plausible mechanism of the role of water in large emission flux changes is provided based on the results of the dynamic temperature experiments and a complementary numerical analysis with a model developed recently by the authors^32^. The mechanism suggests the importance of a global assessment of VOC emissions by continuous measurements of soil VOC emissions of various soil environments and/or predictions of emissions through numerical simulations with thorough consideration of the role of water in dynamic soil environments.

## Materials and methods

### Experimental system

**Figure 1:**
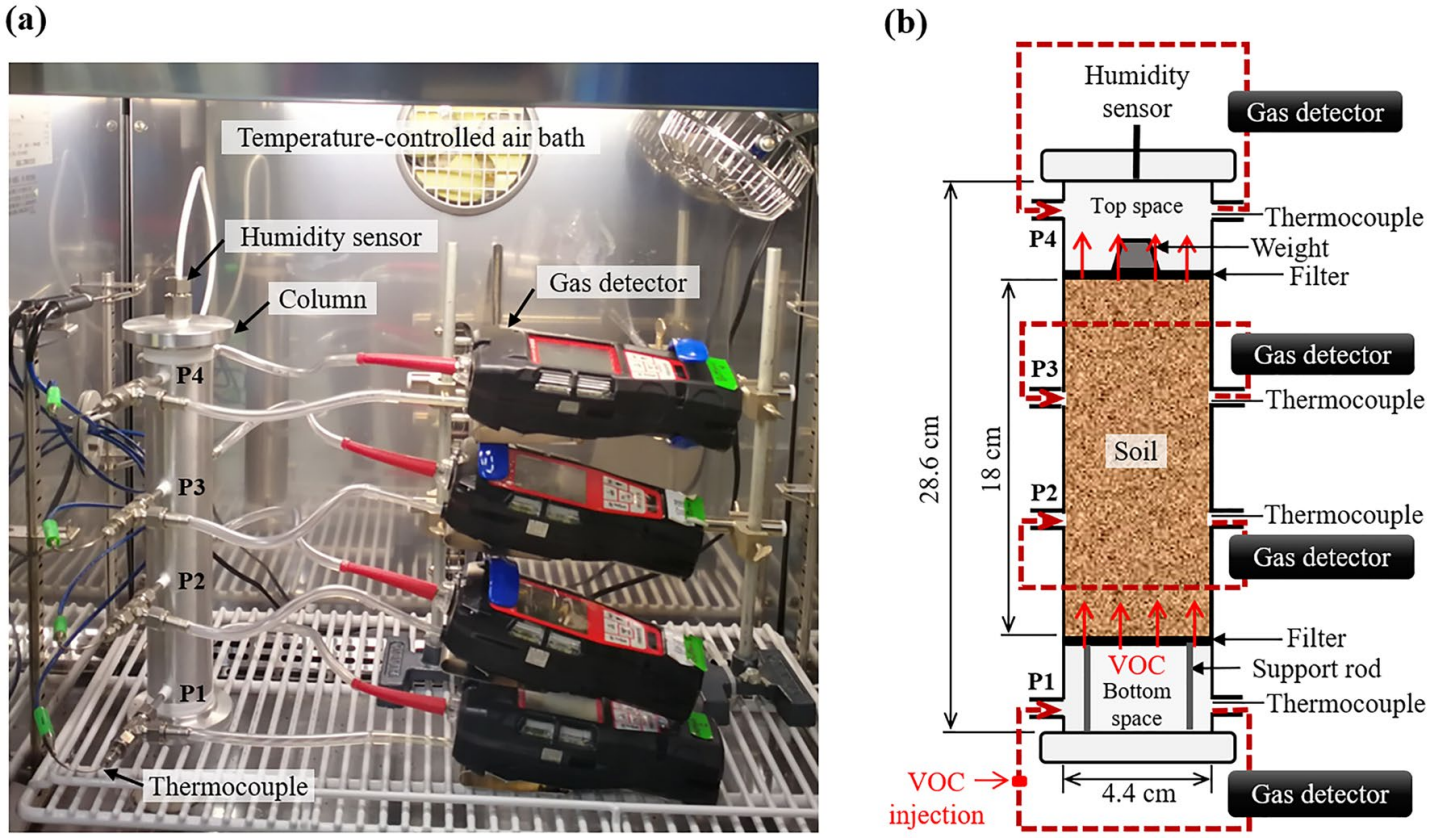
(**a**) Photograph and (**b**) schematic illustration of the experimental system for the vertical vapor-phase diffusive transport of a volatile organic compound (VOC) through a soil sample.

A new experimental system for the vertical vapor-phase diffusive transport of VOCs in a soil sample was developed by using an aluminum column, as shown in Fig. 1. The VOC concentration and temperature could be measured continuously through four ports (P1‒P4 in Fig. 1), and the RH within the column could be measured continuously in the top space. The inner diameter of the column was 4.4 cm and the length was 28.6 cm. A soil sample of approximately 18 cm long with a prescribed amount of water was placed in the center of the column, which was supported at its bottom end by a 1-mm thick stainless-steel-sintered filter and capped at its top end by an additional filter with a 50 g weight to stabilize the soil. It should be noted that all measurements were conducted outside of the soil sample so that the soil pore structure was not disturbed during the experiment.

The four ports (P1‒P4) of the column were monitored by thermocouples and portable gas detectors (GX-6000, Riken Keiki Co., Ltd., Tokyo, Japan). The gas detectors were equipped with a pump to continuously circulate gas between the detector and port; these detectors measured the VOC concentration every 5 min by a photoionization detector. A preliminary experiment revealed that the pump caused no detectable pressure difference (<0.1 kPa) between the bottom and top spaces; therefore, VOC advection caused by pumping was considered unlikely. During the experiment, the column and its sensors and detectors were put in a temperature-controlled air bath that could operate between −15 and 50 °C.

### Experimental procedure

Air-dried natural silica sand (402.3 g; 1.2–2.4 mm grain size) and water (21.2 g; corresponding to 5 wt% water content) were initially packed into the column, where the porosity and gas saturation were 0.43 and 0.82, respectively, in volume fraction. The sand was homogenously packed in the column, as shown by the vertical X-ray computed tomography slice image in Supplementary Figure S2. The image is a representative from several separate images for the entire length of the sand, and was captured at an X-ray tube voltage of 120 kV, X-ray tube current of 150 µA, and voxel size of 40 × 40 × 40 µm by with a microfocus X-ray computed tomography system (ScanXmate-D225RSS270, Comscantecno Co., Ltd., Kanagawa, Japan), where a lighter color in the image corresponds to a higher density. Natural silica sand is produced in Japan and is referred to as Keisha (Takeori Mineral Mining Co., Ltd., Tokyo, Japan). This sand has been utilized widely in Japan for various purposes, and hence its characteristics are well documented^33,34^. Typically, this sand contains ≥90% silica and rarely contains organic impurities. Therefore, influences of sorption and adsorption of VOC by organic materials, which may occur in real subsurface environments, were excluded from the present study. However, the influences of these processes may not necessarily be negligible in real subsurface environments. Hence, such influences should be investigated in future studies.

After the measured temperature at all ports became 25 °C, approximately 5 mL of benzene gas or PCE gas was injected into the bottom space of the column via a gas detector circulation tube with a syringe. A preliminary experiment confirmed that no measurable pressure change (<0.1 kPa) was produced during the injection. The benzene gas and PCE gas were injected after cooling down to approximately 20 °C inside the syringe so that production of liquid-phase VOC within the column was negligible. The benzene and PCE gases were derived from the head-space gases in sealed 10-mL vials containing 0.5 mL of liquid benzene (99.7% purity, Wako Pure Chemical Industry, Ltd., Osaka, Japan) at 60 °C or liquid PCE (99% purity, Wako Pure Chemical Industry, Ltd., Osaka, Japan) at 113 °C, respectively. The boiling point and solubility in water at 25 °C are 80.1 °C and 22.8 mmol/L, respectively, for benzene, and 121 °C and 0.90 mmol/L, respectively, for PCE^35–37^. The diffusion coefficients in air, Henry’s constant of benzene, and PCE for various temperatures are shown in Supplementary Figure S1.

During the experiment, the VOC concentration decreased in the bottom space, whereas it increased in the top space, owing to the upward diffusive transport of the VOCs. The VOC concentration, RH, and temperature were recorded for 120 min under either a constant temperature of 25 °C or sinusoidal temperature variations between 20 and 30 °C (i.e., 25 ± 5 °C). A preliminary experiment confirmed that the temperatures measured at the four ports were very similar, even under temperature variations (Supplementary Figure S3). Consequently, only the mean values are presented in the results. Our previous studies that used similar experimental systems reported that temperature changes at the center of the sand-packed column were delayed only by approximately 5 min when compared to those in the bottom and top spaces^31^. In these previous studies, concentrations were measured at two ports (i.e., P1 and P4 in the present study) using the same gas detectors^30,31^.

The temperature changes between 20 and 30 °C and the decreasing bottom VOC concentration used in the present study were based on a previous field study^13^, which showed that unexpectedly large flux changes occurred with soil temperature changes under these conditions, accompanied by an inverse relationship between VOC flux and temperature. It should be noted that the use of a constant (steady state) VOC concentration in the bottom space, which could have potentially provided clearer and less convoluted results, was considered. However, we found that achieving completely constant concentration was extremely difficult as it required a huge gas reservoir, which was likely to add a notable complexity to our experimental system. We also note that the uniform temperature change within the column replicated the approximately depth-independent temperature variation over a depth range of up to 3 m (except at the soil surface), as reported in the previous field study^13^.

The changes in the VOC concentration measured at P1 of the bottom space and P4 of the top space of the column (Fig. 1) were used to determine the VOC fluxes into and out of the soil sample, respectively, as follows:

1 $${J}_{btm,i}=\frac{{(C}_{P1,i-1}- {C}_{P1,i+1}) \times {10}^{-3}}{22.4}\cdot \frac{273}{273+T}\cdot \frac{{L}_{btm}}{2\Delta t},$$

and

2 $${J}_{top,i}=\frac{{(C}_{P4,i+1}-{ C}_{P4,i-1}) \times {10}^{-3}}{22.4}\cdot \frac{273}{273+T}\cdot \frac{{L}_{top}}{2\Delta t},$$

where *J*_*btm*_,_*i*_ and *J*_*top*_,_*i*_ are the bottom and top VOC fluxes (mol/m^2^/s), respectively, at measurement time *i*, *C*_*P*1,i+1_, *C*_*P*1,i−1_, *C*_*P*4,i+1_, and *C*_*P*4,*i*−1_ are the VOC concentrations (ppm) at P1 and P4 at measurement times *i* + 1 and *i* − 1, respectively, *T* is the temperature (°C), *L*_*btm*_ and *L*_*top*_ are the lengths (m) of the bottom and top spaces, respectively, and Δ*t* is the measurement interval (300 s). In Eqs. (1) and (2), the change in the VOC concentration during 2Δ*t* was used rather than that during Δ*t* to reduce the influence of a nonessential concentration change, which occurs only temporarily. The changes in the RH measured at the top space of the column were used to estimate the water emission to the gas phase in the soil sample, for which positive and negative values corresponded to rates of water evaporation and condensation, respectively, as follows:

3 $${R}_{i}=\left({m}_{i+1}\frac{{h}_{i+1}}{100}-{m}_{i-1}\frac{{h}_{i-1}}{100}\right)\cdot \frac{{V}_{top}}{2\Delta t},$$

where *R*_*i*_ is the water emission (mol/s) at measurement time *i*; *m*_*i*+1_ and *m*_*i*−1_ are the amounts of saturated water vapor (mol/m^3^) at measurement times *i* + 1 and *i* – 1, respectively; *h*_*i*+1_ and *h*_*i*−1_ are the RH (%) at measurement times *i* + 1 and *i* – 1, respectively; and *V*_*top*_ is the volume (m^3^) of the top space.

### Experimental conditions

Three experiments (Runs 1‒3) were performed. Run 1 was conducted using benzene under static temperature (25 °C) to verify the experimental system by confirming no fluctuation in VOC concentration or RH due to the multiple-depth concentration measurement by gas detectors equipped with a pump. Runs 2 and 3 were conducted using benzene and PCE, respectively, under dynamic temperature (20‒30 °C) to examine whether flux changes occurring in these experiments were induced by changes in the vertical concentration gradient of VOCs due to their release from and trapping into water, and/or water evaporation and condensation which contribute to VOC release and trapping, respectively.

### Numerical analysis

The results of Runs 2 and 3 (shown in Sect. “Experimental analysis of flux changes under dynamic temperature”) suggested that the enhanced changes in the emission flux of soil VOC can be attributed to changes in the VOC concentration and its gradient. This can be due to VOC release from/ trapping into water in response to temperature changes and the resulting water evaporation and condensation. Consequently, it was attempted to reproduce such flux changes using a numerical simulation of 1-D (*x*-direction) gas-phase diffusion. A constant effective diffusion coefficient (*D*_*eff*_) was used considering the VOC volatilization from/dissolution into water and the phase change of water. An effective diffusion coefficient is the diffusion coefficient in a porous medium. It is smaller than the diffusion coefficient in air (Supplementary Figure S1a) due to the presence of non-gas-phase materials (e.g., sand and liquid water) that inhibit the gas-phase diffusion process. The effective diffusion coefficient in the present dynamic temperature experiments can be assumed constant because substantial changes in the diffusion coefficient in air and amount of liquid water are unlikely due to small changes at low temperatures (20‒30 °C). Here, a numerical model developed recently by the authors^32^ was used to conduct the simulation for benzene (as a representative VOC), for which the governing equations are:

4 $$\frac{\partial }{\partial t}\left({x}_{g,k}\phi {\rho }_{g}{S}_{g}\right)=\frac{\partial }{\partial x}\left\{{D}_{eff}\frac{\partial }{\partial x}({x}_{g,k}\phi {\rho }_{g}{S}_{g})\right\}+{R}_{vwc,k},$$

and

5 $$\frac{\partial }{\partial t}\left({x}_{w,k}\phi {\rho }_{w}{S}_{w}\right)=-{R}_{vwc,k},$$

where *ϕ* is the porosity (volume fraction); *S*_*g*_ and *S*_*w*_ (= 1 − *S*_*g*_) are the gas and water saturations (volume fraction); *x*_*g*_,_*k*_ and *x*_*w*_,_*k*_ are the mole fractions of component *k* in the gas and liquid water phases (gas phase component: benzene, water, and air; liquid water phase component: benzene and water), respectively; *ρ*_*g*_ and *ρ*_*w*_ are the molar densities (mol/m^3^) of the gas and liquid water phases, respectively; and *R*_*vwc*,*k*_ is the volatilization/condensation or volatilization/dissolution rate for component *k* in the liquid water phase (mol/m^3^/s). Here, *R*_*vwc*,*k*_ for volatilization and condensation (or dissolution) are represented as:

6 $${R}_{vwc,k}={k}_{v, k}\left({x}_{w,k}{P}_{sat,k}-{x}_{g,k}P\right)\left({x}_{w,k}\phi {\rho }_{w}{S}_{w}\right)\,\, \mathrm{at }\,\,{x}_{w,k}{P}_{sat,k}>{x}_{g,k}P,$$

and

7 $${R}_{vwc,k}={k}_{v,k}\left({x}_{w,k}{P}_{sat,k}-{x}_{g,k}P\right)\left({x}_{g,k}\phi {\rho }_{g}{S}_{g}\right)\,\,\mathrm{ at }\,\,{x}_{w,k}{P}_{sat,k}<{x}_{g,k}P,$$

where *k*_*v*,*k*_ is the empirical rate constant (1/Pa/s) for component *k* (1 × 10^–3^ for benzene, 1 × 10^–7^ for water); *P*_*sat*_,_*k*_ is the temperature-dependent saturated vapor pressure (Pa) for component *k*; and *P* is the pressure of the system (Pa). The values of the rate constants were determined experimentally in a previous study^32^. Although the values in the previous and present experiments may not be identical, it is reasonable to assume that the values of rate constants have the same order of magnitude in both studies.

The simulation conditions were similar (but not identical) to those of Run 2, so as to examine the generality of the occurrence of the experimentally observed characteristic flux changes. In the simulation, the soil height was 10 cm, and the porosity and gas saturation of the soil were 0.45 and 0.65, respectively, where the effective diffusion coefficient was 5.4 × 10^–7^ m^2^/s. This soil was divided into ten meshes, and 1-D upward diffusion after introducing benzene at a prescribed mole fraction at the bottom of the soil was simulated by solving the governing equations using the finite difference method with closed boundaries and a 1-min time step. It should be noted that mesh of 1 cm height was used rather than a much finer mesh as each mesh was treated as a porous medium. The temperature was initially 25 °C, and then changed sinusoidally between 20 and 30 °C when relatively slow VOC concentration changes occurred, making it easier to observe the influence of temperature.

## Results and discussion

### Experimental system verification

**Figure 2:**
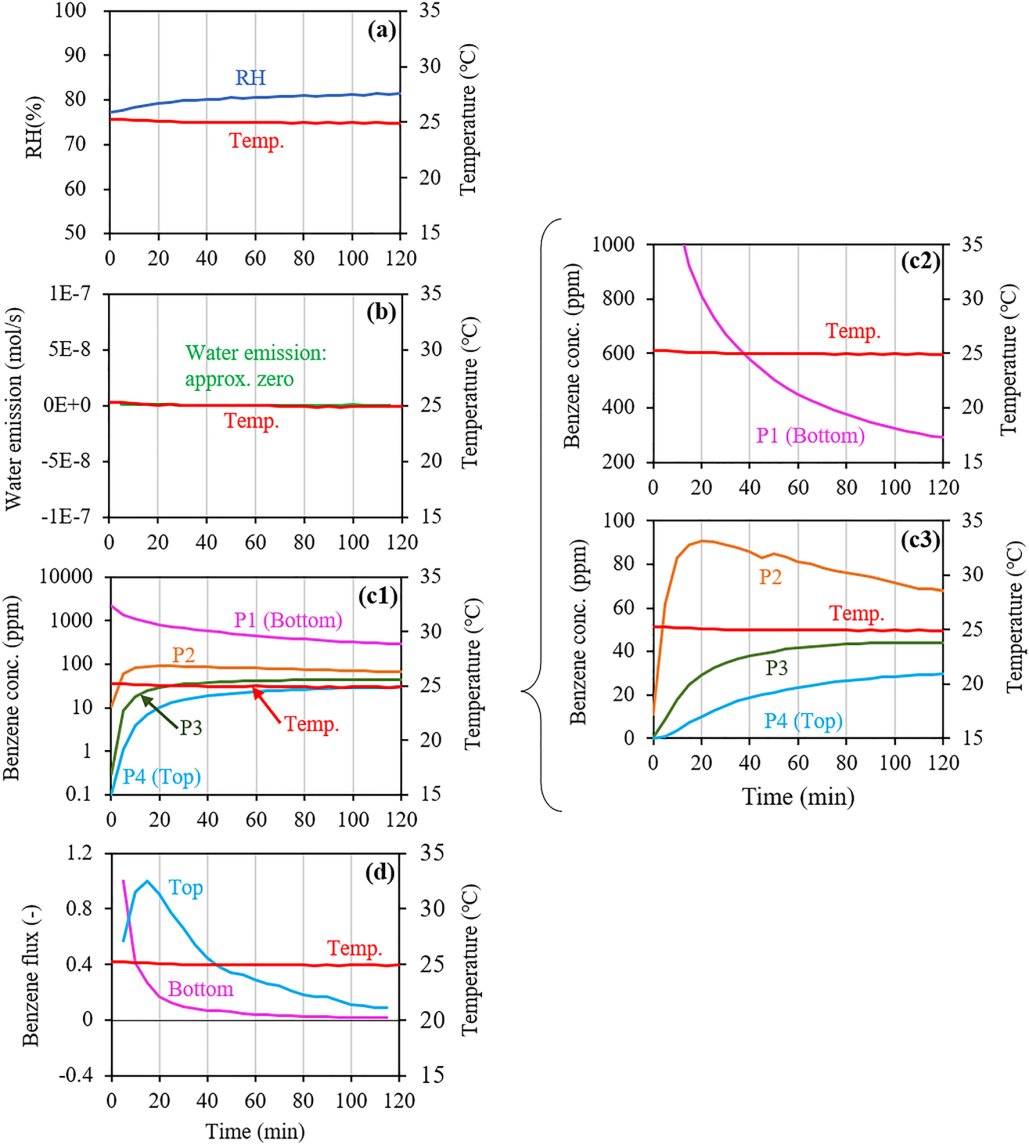
Changes in (**a**) relative humidity (RH) and (**b**) corresponding water emissions, (**c1**‒**c3**) benzene concentrations at the four ports (P1‒P4), and (**d**) bottom and top benzene fluxes estimated from the concentration changes at P1 and P4, respectively, in Run 1 under static temperature of 25 °C. Note that the flux value at each time is represented by a ratio of the observed value at each time to the largest observed value.

Figure 2 shows the changes in the RH and corresponding water emissions measured at the top space, benzene concentrations measured at the four ports (P1‒P4), and the benzene flux estimated for the bottom and top, in the static temperature experiment (Run 1). Note that the flux value at each time shown herein is expressed as the ratio of the observed flux value at each time to the largest value observed during the experiment. In Run 1, the largest values in the bottom and top fluxes were 3.9 × 10^–6^ mol/m^2^/s and 2.4 × 10^–8^ mol/m^2^/s, respectively.

In Run 1, the RH increased slightly and gradually with no substantial fluctuation, owing to the slow evaporation of water over time at a constant temperature (Fig. 2a). Correspondingly, no substantial water emission was observed throughout the experiment (Fig. 2b). The benzene concentration at P1 (bottom) decreased with time due to benzene intrusion into the soil sample (Fig. 2c1). Simultaneously, the benzene concentrations at P2, P3, and P4 (top) increased with time due to benzene diffusion into and emission from the soil sample. Detailed observations of the concentration changes revealed that no substantial fluctuation occurred during the experiment (Fig. 2c2 and c3). As the difference between the bottom and top benzene concentrations generally became smaller with time, the bottom and top fluxes decreased overall (Fig. 2d), providing a mean effective diffusion coefficient (*D*_*eff*_) of 1.3 × 10^–6^ m^2^/s for the entire length of the soil based on Fick’s first law for 1-D (e.g., *x*-direction) diffusion:

8 $$J={-D}_{eff}\frac{dC}{dx},$$

where *J* is the flux (mol/m^2^/s), and *C* is the concentration (mol/m^3^).

Similar changes were observed in our previous studies using similar experimental systems, which involved concentration measurements only at two ports (i.e., P1 and P4 in the present study) using the same gas detectors^30,31^. Thus, the results of Run 1 confirmed the suitability of the experimental system, equipped with two additional measurement ports and gas detectors, to support the objective of the present study.

### Experimental analysis of flux changes under dynamic temperature

**Figure 3:**
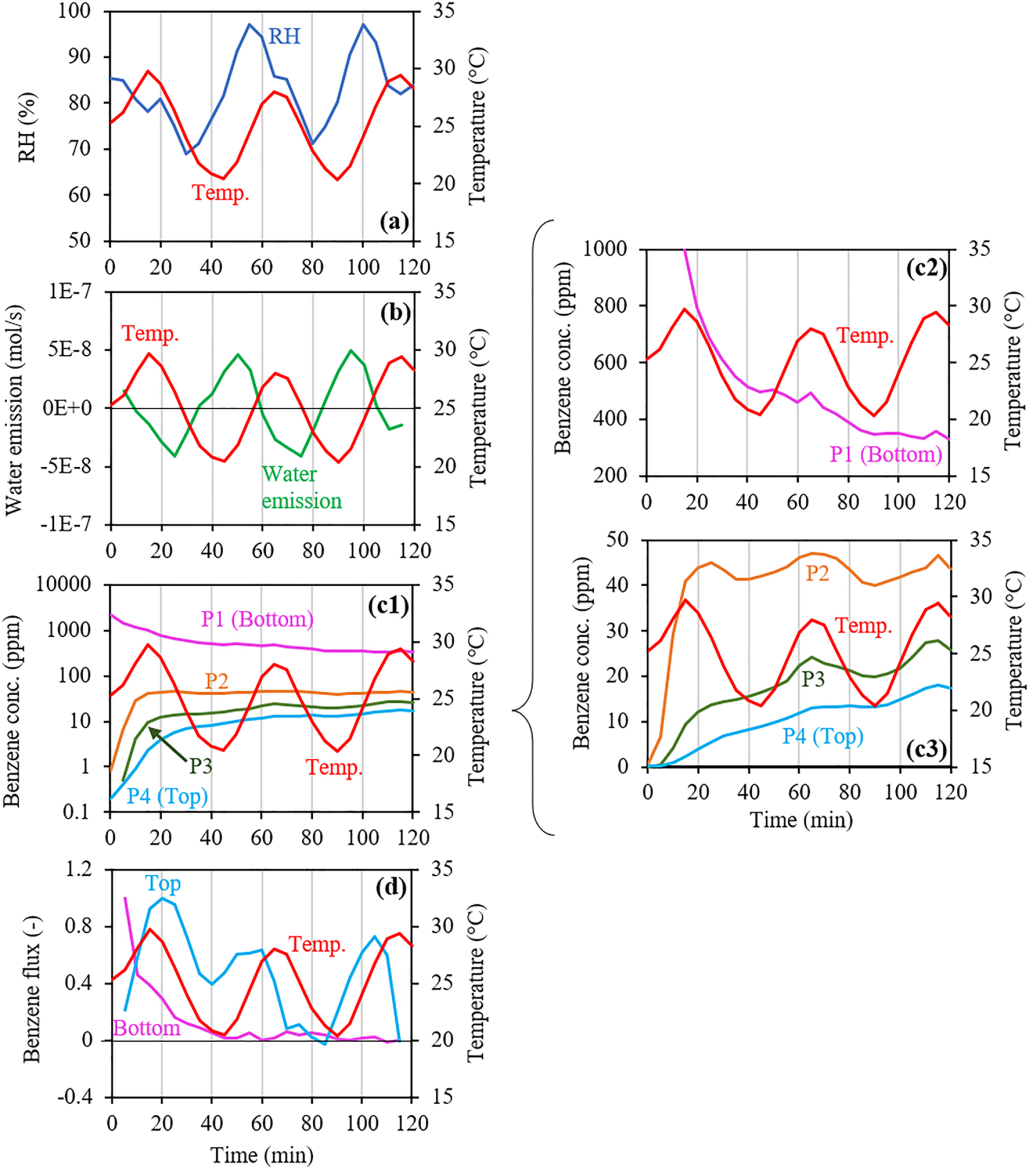
Changes in (**a**) relative humidity (RH) and (**b**) corresponding water emission, (**c1**–**c3**) benzene concentrations at the four ports (P1–P4), and (**d**) bottom and top benzene fluxes estimated from the concentration changes at P1 and P4, respectively, in Run 2 under dynamic temperature (20‒30 °C). Note that the flux value at each time is represented by a ratio of the observed value at each time to the largest observed value.

Figure 3 shows the changes in the RH, water emission, benzene concentrations at P1‒P4, and bottom and top benzene fluxes in the dynamic temperature experiment (Run 2), where the largest absolute values in the bottom and top fluxes were 3.6 × 10^–6^ mol/m^2^/s and 1.2 × 10^–8^ mol/m^2^/s, respectively. For this experiment, the only difference between the experimental conditions and those in Run 1 was a change in the temperature condition to dynamic temperature.

In Run 2, substantial changes in RH occurred during the temperature changes due to the temperature-dependent amount of saturated water vapor and water phase changes, i.e., evaporation and condensation (Fig. 3a). Based on the RH data, a largely negative relationship between changes in water emission and temperature was evident (Fig. 3b), showing both positive and negative emissions, corresponding to water evaporation and condensation, respectively. Overall changes in benzene concentrations at P1‒P4 were similar to those in Run 1 (Fig. 3c1); however, detailed observations revealed a largely positive relationship with temperature changes at all depths (Fig. 3c2 and c3). The changes in the top flux were related positively to temperature (Fig. 3d). However, based on the temperature dependency of the diffusion coefficients of benzene in air (Supplementary Figure S1a), unexpected changes occurred in the top flux. For instance, the top flux reached a negative value at 85 min and then increased by more than an order of magnitude until 105 min. Moreover, the bottom flux exhibited unexpected changes, whereby it started to increase at 60 min, reaching a peak at approximately 80 min, during which the top flux decreased to make the opposite reach a peak under decreasing temperature. These flux changes demonstrated occurrences of enhanced flux changes and an inverse relationship between changes in flux and temperature under dynamic temperature, as suggested and reported in previous studies^13,18,30,31^.

**Figure 4:**
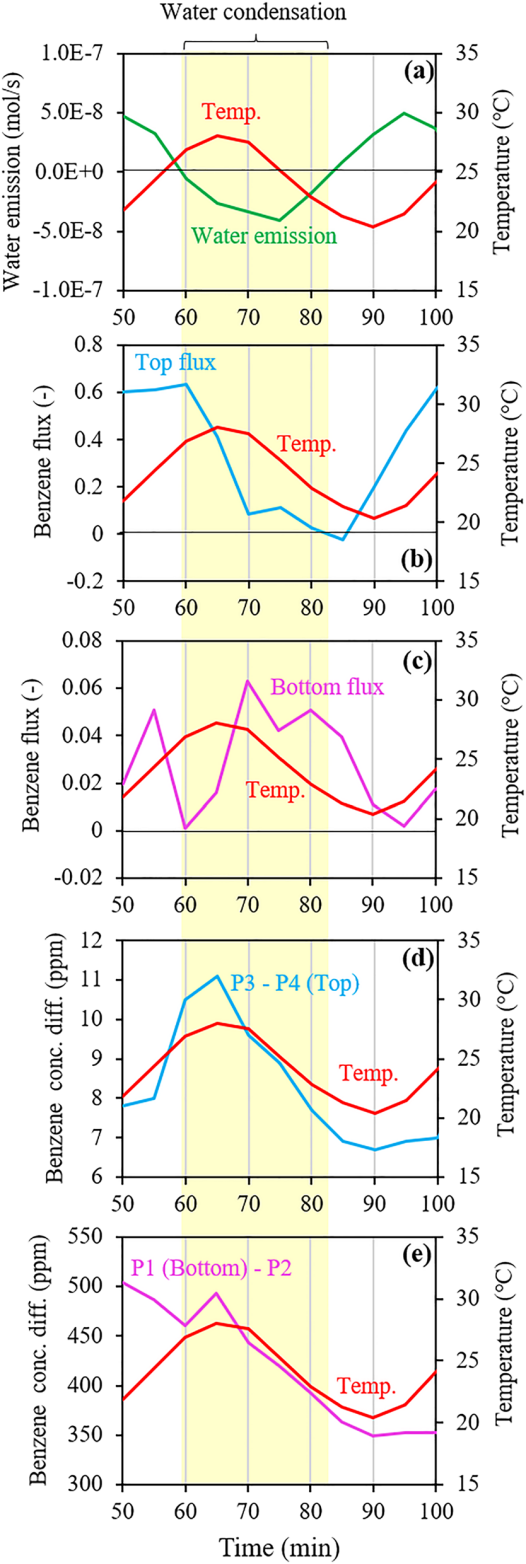
Changes in (**a**) water emissions; (**b**) top and (**c**) bottom benzene fluxes; and (**d** and **e**) upper and lower benzene concentration differences during 50–100 min in Run 2 under dynamic temperature (20‒30 °C). Note that the flux value at each time is represented by a ratio of the observed value at each time to the largest observed value during the entire experimental duration.

The flux changes in response to temperature changes were identified more easily after approximately 50 min because the changes before it were large, caused by the larger difference between the bottom and top concentrations (i.e., the concentration gradient d*C*/d*x* in Eq. (8)). Consequently, changes in the top and bottom fluxes during 50‒100 min are summarized in Fig. 4b, c, respectively. Additionally, in Fig. 4, the time during which water condensation proceeded (Fig. 4a) is highlighted by the yellow band.

As stated earlier, changes in the top flux were largely related positively to temperature changes. However, this detailed investigation based on Fig. 4 implied that the phase changes of water in addition to temperature changes had a substantial impact on the flux changes. For instance, the top flux started to decrease at 60 min (Fig. 4b) under an increasing temperature, around which negative water emissions (i.e., water condensation) occurred (Fig. 4a). Additionally, the top flux showed a negative peak at approximately 70‒85 min, during which water emissions showed a peak whereas the temperature continued to decrease. Simultaneously, the bottom flux started to increase at 60 min and showed a peak at approximately 70‒85 min (Fig. 4c). Consequently, both the phase change of water and the temperature change may have affected the flux change.

Based on Eq. (8), the top and bottom flux changes may have been caused by changes in the concentration gradient in the soil sample, as hypothesized in the introduction. Figure 4 also compares changes in the top and bottom fluxes (Fig. 4b, c), and concentration differences in the upper and lower parts of the soil sample (Fig. 4d, e) during 50‒100 min in Run 2. The upper concentration difference was calculated as the difference between the P3 and P4 concentrations, and the lower concentration difference was calculated as the difference between the P1 and P2 concentrations.

The upper concentration difference showed a decreasing trend during 60‒85 min (Fig. 4d), during which the top flux also decreased (Fig. 4b), which is consistent with the hypothesis. However, changes in the concentration gradient could not quantitatively explain the observed flux changes. For instance, during 60‒85 min, the difference between the minimum and maximum values was less than twofold for the concentration difference, whereas it exceeded an order of magnitude for the flux. Moreover, the relationship between the changes in the lower concentration difference and bottom flux was unclear (Fig. 4c, e). Consequently, the hypothesis that changes in the concentration gradient cause the enhanced flux changes and the inverse relationship between changes in flux and temperature, was demonstrated only in part, which suggests that changes in the concentration gradient that were responsible for the bottom and top flux changes occurred more locally.

**Figure 5:**
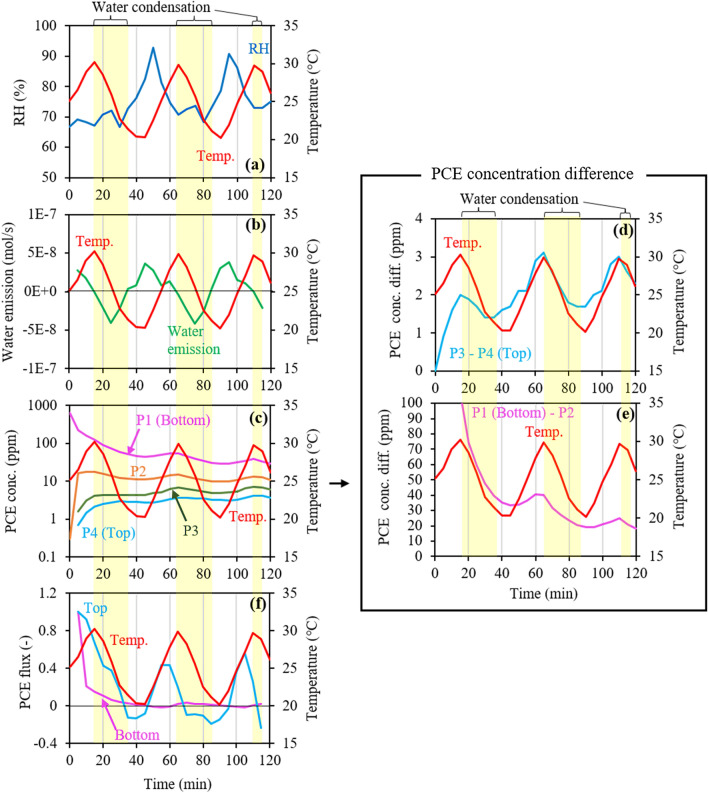
Changes in (**a**) relative humidity (RH) and (**b**) corresponding water emissions, (**c**) tetrachloroethylene (PCE) concentrations at the four ports (P1‒P4), (**d**, **e**) corresponding upper and lower concentration differences, and (**f**) bottom and top PCE fluxes estimated from the concentration changes at P1 and P4, respectively, in Run 3 under dynamic temperature (20‒30 °C). Note that the flux value at each time is represented by a ratio of the observed value at each time to the largest observed value.

Figure 5 shows the changes in the RH, water emission, PCE concentrations at P1‒P4 with corresponding lower and upper concentration differences, and bottom and top PCE fluxes in the dynamic temperature experiment (Run 3), where the largest absolute values in the bottom and top fluxes were 1.6 × 10^–6^ mol/m^2^/s and 5.5 × 10^–9^ mol/m^2^/s, respectively. For this experiment, the only difference between the experimental conditions and those in Run 2 was essentially the change in the VOC type to PCE, having a stronger temperature dependency in volatility (Supplementary Figure S1b). In general, the results for Run 3 were qualitatively similar to those in Run 2. However, the changes in concentration with temperature changes were more evident, and the top flux changes were more intense, whereby a much larger negative flux occurred. This is consistent with the findings of a previous study^31^ that flux changes in response to temperature changes were larger for TCE than benzene, whereby TCE has a stronger temperature dependency in volatility (Supplementary Figure S1b). Consequently, the Run 3 results strongly supported the hypothesis that VOC release from and trapping into water, and/or water evaporation and condensation, change the VOC concentration to alter its vertical gradient, which finally causes larger flux changes and an inverse relationship between flux and temperature changes.

### Role of water in flux changes under dynamic temperature

**Figure 6:**
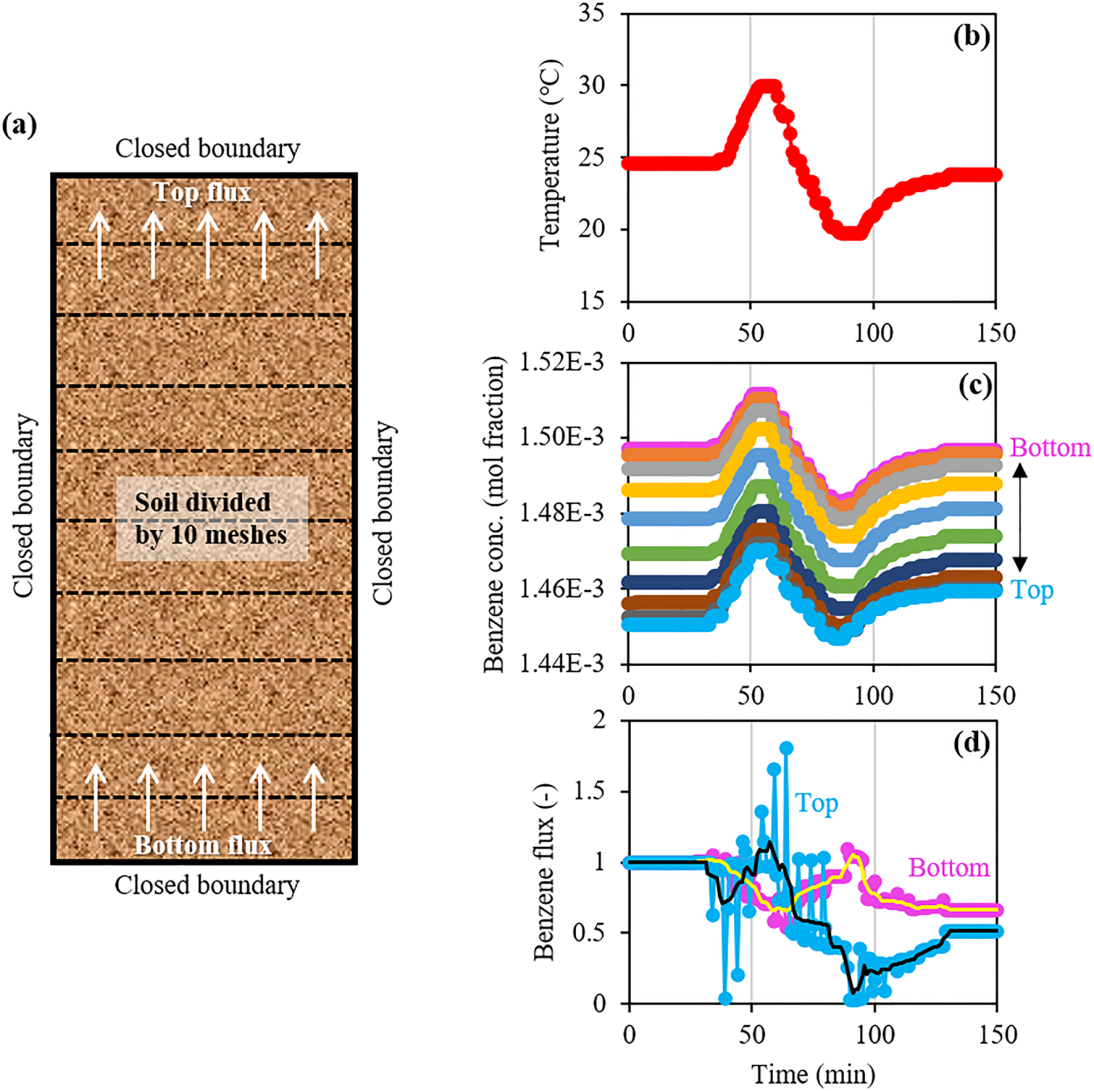
(**a**) Model setup, changes in (**b**) temperature, (**c**) benzene concentration at each mesh, and (**d**) bottom and top fluxes in the numerical simulation. Note that the smoothed flux changes were obtained by 5-point (5-min) adjacent averaging.

Based on the results of the dynamic temperature experiments in Runs 2 and 3, the enhanced changes in the emission flux of soil VOC may have been attributed to changes in the VOC concentration and its gradient due to VOC release from/ trapping into water in response to temperature changes and the resulting water evaporation and condensation. If this is true, such flux changes can be reproduced by the numerical simulation of 1-D gas-phase diffusion with a constant effective diffusion coefficient considering the VOC volatilization from/dissolution into water and the phase change of water. Figure 6 shows the changes in temperature, mole fraction of benzene in the gas phase at each mesh, and top and bottom fluxes of benzene in the gas phase based on Fick’s first law (Eq. (8)) during the time of the sinusoidal temperature change. It should be noted that the flux value was the ratio of the flux value at each time step to the value prior to changing the temperature. Moreover, in Fig. 6d for the fluxes, smoothed lines derived by 5-point (5-min) adjacent averaging of flux data are also shown to compare them with the results in Run 2 with a 5-min data interval. Even though the concentration at each mesh changed smoothly (Fig. 6c), the concentration difference between the adjacent meshes, which was used to calculate the flux (Eq. 8), did not change smoothly. This resulted in rapid fluctuations in the original flux data.

At all meshes in the model (Fig. 6a), the benzene concentration changed in response to temperature (Fig. 6b, c), which was consistent with the largely positive relationship between concentration and temperature changes at all depths in Run 2 (Fig. 3c2 and c3). As for the top and bottom fluxes (Fig. 6a), the changes in the top flux in the model were positively related to temperature changes, the difference between the minimum and maximum fluxes was at least an order of magnitude, and the changes in bottom flux were inversely related to temperature changes (Fig. 6b, d), which were consistent with the flux changes (Fig. 4b, c). Because the effective diffusion coefficient was independent of temperature in this simulation, the bottom and top flux changes were caused only by changes in the concentration gradient near the top and bottom, respectively. Thus, this study clarified that the enhanced changes in the emission flux of soil VOCs can be caused by changes in the VOC concentration gradient due to the concentration changes caused by VOC release from/trapping into water in response to temperature changes.

Based on the mechanisms or role of water in the enhanced emission flux changes, such changes may occur in any soil environment where water exists, with some site-specific characteristics. Consequently, continuous measurements of soil VOC emissions for various soil environments, and/or predictions of emissions through numerical simulations considering the role of water in dynamic soil environments^32^, are essential for a global assessment of the impacts of soil VOC emissions on atmospheric conditions and climate.

## Conclusions

Understanding the transport behavior of VOCs in near-surface soils is quite important because of the potential influences of soil VOC emissions on atmospheric conditions and climate. However, the mechanisms of much larger changes in the VOC emission flux under dynamic temperatures compared to those expected from the temperature dependency of the diffusion coefficients of VOCs in the air have been poorly understood, although water in soil has been suggested to play a substantial role. The results of the experiment with a novel system developed in this study and the complementary numerical simulation with a new model developed in a recent study, clarified, for the first time, that the enhanced changes in emission flux may occur as a result of changes in the VOC concentration gradient due to concentration changes induced by VOC release from/trapping into water in response to temperature changes. The changes in emission flux can exceed an order of magnitude with a temperature change of 10 °C, and such enhanced changes may occur with site-specific characteristics in any soil environment where water exists. Consequently, to quantitatively understand the impacts of soil VOC emissions on atmospheric conditions and climate, a global assessment of VOC emissions requires continuous measurements of soil VOC emissions for various soil environments and/or predictions of emissions through numerical simulations, considering the role of water in dynamic soil environments.

## Supplementary Information

Supplementary Information.

## Data Availability

The data that support the findings of this study are available from the corresponding author on reasonable request.
