# Supplementary material for: Role of water in unexpectedly large changes in emission flux of volatile organic compounds in soils under dynamic temperature conditions
Source: Sci Rep. 2022 Mar 15;12:4418. doi: 10.1038/s41598-022-08270-5 (PMC8924235; doi:10.1038/s41598-022-08270-5)
Supplement: Supplementary file 1 — Supplementary Information. [file 41598_2022_8270_MOESM1_ESM.docx]

Supplementary Information

**Role of water in unexpectedly large changes in emission flux of volatile organic compounds in soils under dynamic temperature conditions**

Asma Akter Parlin^1,^*, Monami Kondo^1,^*, Noriaki Watanabe^1,^*, Kengo Nakamura^1^, Jiajie Wang^1^, Yasuhide Sakamoto^2^, and Takeshi Komai^1^

^1^ Department of Environmental Studies for Advanced Society, Graduate School of Environmental Studies, Tohoku University, Sendai 9808579, Japan

^2^ National Institute of Advanced Industrial Science and Technology (AIST), Tsukuba 3058567, Japan

*Corresponding authors.

E-mail addresses: asma.akter.parlin.r6@dc.tohoku.ac.jp (A. A. P.), monami.kondo.p3@dc.tohoku.ac.jp (M. K.), noriaki.watanabe.e6@tohoku.ac.jp (N. W.)

Tel & Fax: +81-22-795-7384 (A. A. P, M. K., and N. W.)

**Supplementary Figure S1. Temperature dependence of (a) diffusion coefficients in the air and (b) dimensionless Henry’s constants of trichloroethylene (TCE), tetrachloroethylene (PCE), and benzene [38].**

**Supplementary Figure S2. Representative X-ray computed tomography slice image of a portion of the column containing sand.**

**Supplementary Figure S3. Measured temperatures at the four ports (P1‒P4) under sinusoidal temperature variations between 20 and 30 °C.**
